# Diabetic Foot Ulcers Detection Model Using a Hybrid Convolutional Neural Networks–Vision Transformers

**DOI:** 10.3390/diagnostics15060736

**Published:** 2025-03-15

**Authors:** Abdul Rahaman Wahab Sait, Ramprasad Nagaraj

**Affiliations:** 1 Department of Archives and Communication, Center of Documentation and Administrative Communication, King Faisal University, P.O. Box 400, Hofuf 31982, Al-Ahsa, Saudi Arabia; 2 Department of Biochemistry, S S Hospital, S S Institute of Medical Sciences & Research Centre, Rajiv Gandhi University of Health Sciences, Davangere 577005, Karnataka, India; ramprasad7u@gmail.com

**Keywords:** deep learning, vision transformers, feature fusion, diabetic mellitus, foot ulcers, ischemia, Kolmogorov–Arnold networks

## Abstract

**Background:** Diabetic foot ulcers (DFUs) are severe and common complications of diabetes. Early and accurate DFUs classification is essential for effective treatment and prevention of severe complications. The existing DFUs classification methods have certain limitations, including limited performance, poor generalization, and lack of interpretability, restricting their use in clinical settings. **Objectives:** To overcome these limitations, this study proposes an innovative model to achieve robust and interpretable DFUs classification. **Methodology:** The proposed DFUs classification integrates MobileNet V3-SWIN, LeViT-Peformer, Tensor-based feature fusion, and ensemble splines-based Kolmogorov–Arnold Networks (KANs) with Shapley Additive exPlanations (SHAP) values to classify DFUs severities into ischemia and infection classes. In order to train and generalize the proposed model, the authors utilized the DFUs challenge (DFUC) 2021 and 2020 datasets. **Findings:** The proposed model achieved state-of-the-art performance, outperforming the existing approaches by obtaining an average accuracy of 98.7%, precision of 97.3%, recall of 97.4%, and F1-score of 97.3% on DFUC 2021. On DFUC 2020, it maintained a robust generalization accuracy of 96.9%, demonstrating superiority over standalone and baseline models. The study findings have significant implications for research and clinical practice. The findings offer an effective platform for scalable and explainable automated DFUs treatment and management, improving patient outcomes and clinical practices.

## 1. Introduction

A significant consequence of diabetes mellitus, diabetic foot ulcers (DFUs) affect millions of individuals across the globe [1]. These ulcers pose a significant public health risk, leading to morbidity, amputations, and mortality. Factors including neuropathy, ischemia, and infection delay the healing process, resulting in the formation of these ulcers [2]. DFUs have a substantial impact on individuals’ quality of life [3]. Individuals with chronic wounds generally experience intense pain, restricted movement, and a lack of social interaction [4]. These wounds require extended medical consultations, dressing changes, and occasionally surgery interventions. Due to their limited physical ability and dependency on caretakers, individuals encounter depression and anxiety [5]. Hospitalization or amputations may increase the length of stay and cause substantial healthcare expenses.

Effective DFU therapy requires timely and precise ischemia and infection identification [6]. Traditionally, DFUs diagnosis relied on laboratory testing, imaging, and clinical evaluations [7]. Clinicians identify infections using clinical symptoms, bacterial cultures, and inflammatory markers, whereas perfusion levels determine ischemia. The dependence on clinical knowledge renders these procedures laborious, subjective, and inconsistent. Imaging modalities, including thermal, hyperspectral, and fluorescence imaging have gained traction in diagnosing DFUs in the initial stages [8]. These modalities offer detailed insights into blood flow, tissue composition, and infection biomarkers [9]. However, extensive implementation costs and a steep learning curve for healthcare professionals prevent the widespread adoption of these modalities. In contrast, photographic images capture visible wound features, including size, shape, and discoloration, with sufficient clarity [10]. These features can be shared for telemedicine consultations, supporting early DFUs diagnosis.

In the absence of prompt medical attention, ulcers have the potential to penetrate deep into soft tissue, tendons, and potentially bone, elevating the likelihood of developing osteomyelitis and systemic infections leading to the amputation of the lower extremities [10]. By using an artificial intelligence-powered DFUs detection system, healthcare personnel may initiate debridement and infection management in the early stages, preventing severe consequences [11]. The advent of deep learning (DL) techniques has enabled automated DFUs detection, minimizing human errors. These models can capture crucial features associated with ischemia and infection classes using photographic images [11]. They are ideal for high-throughput diagnostics, handling large volumes of images in limited time [12]. Convolutional neural networks (CNNs) and vision transformers (ViTs) are robust DL architectures, playing a significant role in extracting features from the images [13]. Each architecture has the potential to offer unique DFUs features, contributing to reliable DFUs detection. CNNs use convolution layers to capture the size, shape, and texture of ulcers with surrounding skin abnormalities [14]. The hierarchical structure detects subtle features of ischemia and infection. Similarly, ViTs identify global relationships within an image [15]. These models process an image as a sequence of patches. The overall condition and spatial relationships between multiple ulcers can be extracted through the ViTs architecture. The inherent adaptability of ViTs enables model interpretability, highlighting regions influencing their predictions [16]. Although CNNs and ViTs architectures demonstrate remarkable outcomes, they fail to provide deeper insights into their decision-making processes [17]. Accurate DFUs detection and classification requires diverse meaningful features [18]. The feature fusion technique enriches the data representation. The absence of reliable feature fusion techniques integrating local and global features leads to suboptimal classification outcomes. The lack of model interpretability and inability to combine the features of multiple architectures cause challenges in identifying ischemia and infection images [19].

The ensemble learning (EL) approach combines the predictions of multiple models [20]. It reduces the risk of overfitting the training data. It enhances the model’s generalization capability [21]. In the context of DFUs classification, an individual classification model may struggle to maintain a trade-off between specificity and sensitivity. Combining feature fusion and EL approaches can significantly improve classification performance. These features motivated the authors to develop an EL-based DFUs classification model. The proposed model integrates the advanced feature extraction and EL approaches to identify DFUs in the initial stages. In addition, it offers interpretable outcomes to support clinicians in making effective decisions. In this study, the authors provide the following unique contributions to the DFUs detection and classification literature:

1. Hybrid feature extraction framework using the potentials of CNNs and ViTs:

The proposed framework leverages the strengths of MobileNet V3-SWIN and LeViT-Performer models to extract fine-grained localized features and spatial arrangement of ulcers and healthy tissues. It addresses the limitations of individual approaches by developing a robust and comprehensive feature extraction process.

2. Tensor fusion technique-based critical feature identification:

An innovative feature fusion is introduced to combine key features associated with ischemia and infection images. By leveraging the potential of tensor fusion techniques, the proposed feature fusion overcomes the limitations of existing techniques. In addition, it preserves the integrity of diverse features.

3. Ensembled splines-driven Kolmogorov–Arnold Networks (KANs)-based DFUs classification.

The proposed study offers a technique to ensemble the outcomes of spline functions within the KANs. It enhances the interpretability and classification performance. The KANs-based classification uses Shapley additive explanations (SHAP) to build Explainable AI, providing insights into the classification process.

The remaining part of this study is structured into four key sections, presenting a comprehensive understanding of the development and evaluation of an interpretable DFUs classification model. Section 2 outlines the data preparation and architectural design of the proposed model. It reveals the hybrid feature extraction and KANs-based classification approaches. The study findings are outlined in Section 3. Section 4 contains the interpretation of the findings and emphasizes the model’s contributions to DFUs classification. It covers the challenges and future model improvements. Lastly, Section 5 summarizes the study’s outcomes, underscoring the potential clinical impact of the proposed DFUs classification.

## 2. Materials and Methods

**Figure 1:**
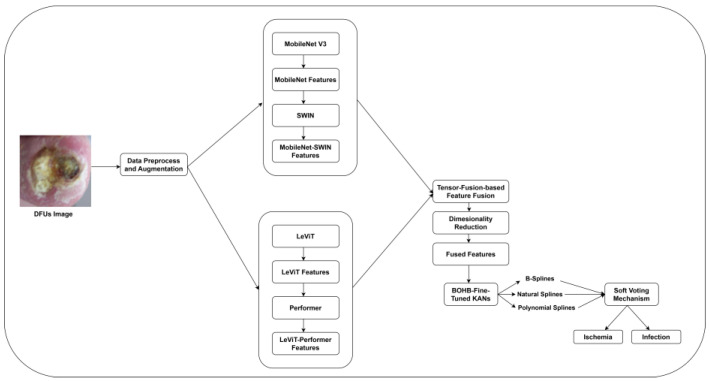
The proposed DFUs classification.

This study introduces a model integrating hybrid feature extraction, tensor fusion-based feature fusion, and KANs-based DFUs classification. The synergy of MobileNet V3-SWIN and LeViT-Performer overcomes the shortcomings of individual CNNs and ViTs-based feature extraction models. To optimally integrate the diverse DFUs features, the authors employed tensor fusion-based feature fusion. This approach was used to enhance the feature representation and improve model robustness. The KANs-based classifier with SHAP values decomposes complex decision boundaries into smooth and explainable sub-functions, achieving reliable and interpretable classification. The proposed research methodology is outlined in Figure 1.

### 2.1. Data Acquisition and Preparation

**Figure 2:**
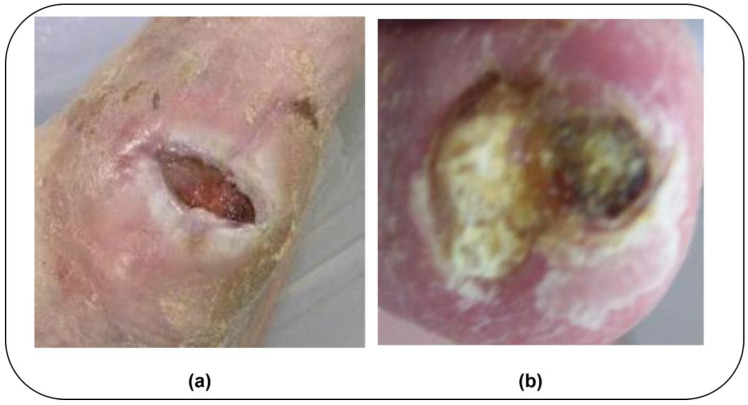
(**a**) Infection and (**b**) Ischemia.

The effectiveness of DL-based DFUs classification depends on the quality and diversity of the dataset. In this study, the authors utilized the diabetic foot ulcer challenge (DFUC) 2021 and DFUC 2020 datasets [22] to develop a robust and interpretable model. They selected these datasets due to high-quality annotations, balanced class distributions, and diverse image conditions. The images were captured using three digital cameras, including Kodak DX4560, Nikon D3300, and Nikon Coolpix P100. Proper authorization was obtained from the dataset providers to use the foot images for this study. DFUC 2021 [22] contains 15,683 images, whereas DFUC 2020 [22] includes 4000 foot images. These are designed for DFUs classification. They cover ulcer images collected over different time frames. As a result, the proposed model can understand the temporal evolution of DFUs, distinguishing between early-stage and chronic ulcers. It can evaluate the progression of the disease using the dataset’s time-series characteristics. Expert wound care specialists clinically evaluated the DFUC datasets. They manually annotated and classified the ulcers based on medical guidelines and diagnostic criteria. The datasets serve as a gold standard benchmark for evaluating DL-based DFUs classification models. By leveraging the DFUC 2021 and DFUC 2020 longitudinal datasets, the proposed model can support dynamic, personalized, and clinically relevant ulcer management in real-world settings. Figure 2a,b show the sample infection and ischemia images of the DFUC 2021 dataset. The use of multiple datasets offers an environment for comprehensive and unbiased evaluation. Table 1 reveals the key features of the datasets.

Although CNNs and ViTs are designed for robust feature extraction using the raw images, data preprocessing remains crucial to guarantee generalization and stability in DFUs classification. The authors standardized the images to 224 × 224 pixels. Mean subtraction and standard deviation scaling were used to scale the pixel intensity values between 0 and 1. Additionally, Gaussian filtering was employed to smoothen artifacts in order to overcome unnecessary distortions in feature representations. The authors used the LAB color space conversion technique to enhance ischemia detection. To improve model generalization, the authors employed data augmentation techniques. The data augmentation techniques introduce controlled perturbations into the dataset to train the model to recognize perturbation patterns and adjust accordingly. Geometric transformations, including random rotations, horizontal flips, and perspective transformations, were used to train the model to learn orientation-invariant features. Random rotation supports the model in identifying ulcers from different orientations. Horizontal and vertical flipping addresses variability in foot positioning. It assists the model in maintaining stability across real-time images. Random clipping was performed to prevent the model from being dependent on specific ulcer positions within the image. Color augmentations, including brightness, contrast, saturation, and hue adjustments, were used to simulate variations in clinical lighting conditions. The simulation of real-world distortions can improve the model’s resilience to noise, occlusions, and imaging inconsistencies. In order to generate synthetic ulcer images, the authors used a cycle-consistent generative adversarial network (CycleGAN). CycleGAN applies image-to-image translation, allowing the transformation of images between domains without requiring paired training data. To counteract the class imbalances, the authors selectively applyed data augmentation to minority classes while maintaining relatively unchanged non-infected ulcer images.

### 2.2. MobileNet V3-SWIN Feature Extraction

**Figure 3:**
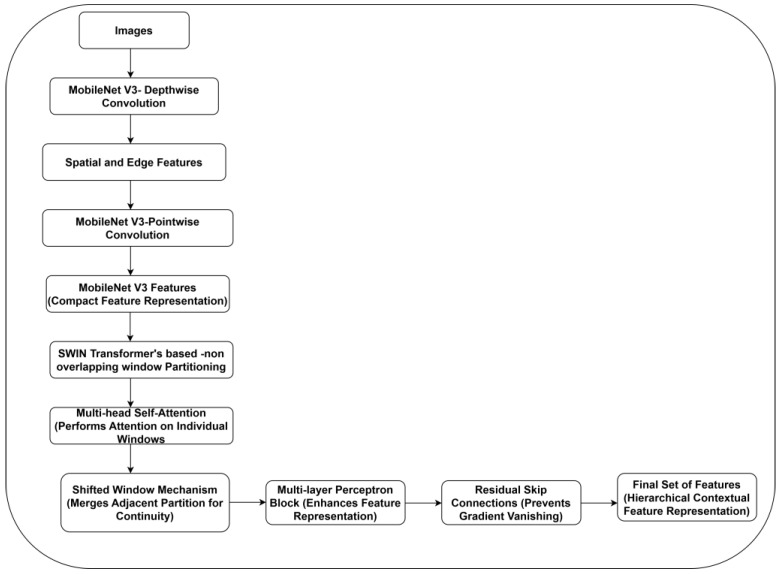
MobileNet V3-SWIN-based feature extraction.

MobileNet V3 [23] is an optimized CNN backbone using depthwise separable convolutions to extract the fine-grained texture, ulcer boundaries, and local tissue characteristics. The effective spatial encoding technique captures ulcer-specific features better than a standard CNN layer. SWIN transformer typically processes the image with a convolutional layer. This layer is limited in its ability to capture complex textures, edges, and fine-grained patterns of ulcers. Thus, the authors replaced the SWIN transformer’s initial convolutional stem with the MobileNet V3 backbone. Figure 3 illustrates the feature extraction process using the synergy of the MobileNet V3-SWIN model.

The MobileNet V3 model facilitates early-stage feature extraction while the SWIN transformer hierarchically handles the extracted feature maps. Equation (1) shows the feature extraction approach using the MobileNet V3 model.

(1) $$F_{M}=\sigma \left(W_{d}*X\right)⊙W_{P}$$

where $F_{M}$ is the MobileNet V3 feature, $W_{d}$ is the depthwise convolution kernel, $W_{P}$ is the pointwise convolution kernel, σ is the hard-swish activation, ∗ is the matrix multiplication, and ⊙ is the element-wise multiplication.

SWIN divides the extracted feature maps into small non-overlapping windows and applies self-attention within each window. Without substantial memory, it refines high-dimensional features. Equation (2) outlines the process of feature representation using multi-head self-attention (MSA).

(2) $${FR}^{l}=MSA\left(Q,K,V\right)+{FR}^{l-1}$$

where$\;{FR}^{l}$ is the feature representation at layer *l*, and $MSA\left(Q,K,V\right)$ is computed using Equation (3).

(3) $$MSA\left(Q,K,V\right)=Softmax\left(\frac{{QK}^{T}}{\sqrt{d}}\right)V\;$$

where Q,K,and V are the query, key, and value matrices, and *d* is the scaling factor.

SWIN [24] employs the shifted windows function in the subsequent layers, maintaining continuity in feature representation. By leveraging multiple transformer blocks, the final global feature representation ($F_{S}$) is obtained, encapsulating high-level spatial relationships and structural abnormalities over the DFUs region. Gradient SHAP values are used to highlight the feature contributions. Although SWIN guarantees optimal feature utilization, the fine-grained local details of the MobileNet V3 may not be preserved. Thus, the authors retained the MobileNet V3 features and fused them with SWIN hierarchical features using the tensor fusion-based feature fusion.

### 2.3. LeViT-Performer Feature Extraction

**Figure 4:**
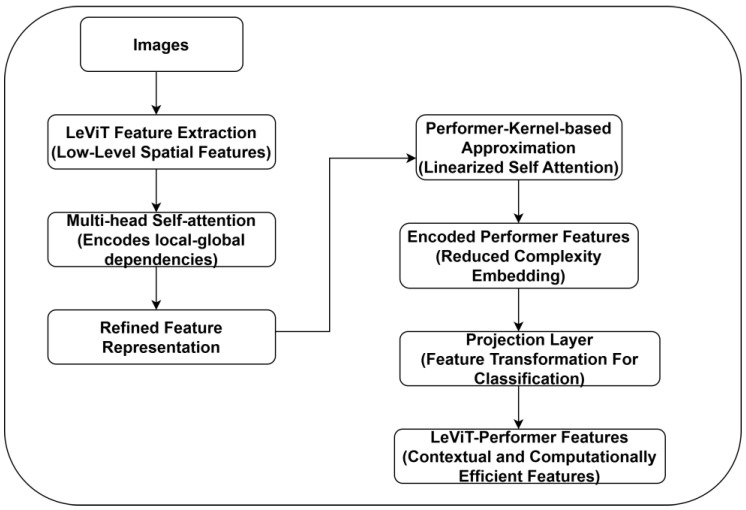
LeViT-Performer-based Feature Extraction.

To extract diverse DFUs features, the authors employed a hybrid ViTs-based feature extraction. They combined the potential of LeViT [25] and Performer [26] models. By leveraging this strategy, fine details such as ulcer textures, boundaries, and infection patterns with global structural dependencies, including ulcer distribution across foot regions, are extracted. Figure 4 reveals the recommended LeViT-Performer model.

Initially, LeViT captures fine details using its CNN-based embedding. Equation (4) outlines the feature extraction process.

(4) $$F_{L}=\sigma \left(W_{c}*X\right)+b$$

where $F_{L}$ is the LeViT features, $X\leftarrow \;R^{H\times W\times C}$ is the image, $W_{c}$ is the convolutional weight matrix, ∗ represents the convolutional operation, R is the image tensor with height (*H*), weight (*W*), and channels (*C*), and σ is the GELU activation function.

To refine the extracted features, LeViT uses MSA. This approach generates the ulcer-specific textual variations. However, it lacks the ability to effectively model long-range feature dependencies. To overcome this limitation, the Performer transformer is used to represent features with global ulcer patterns. The Kernel-based approximation is employed to encode the global features. The transformer SHAP value is used to identify the critical features using their weights. The projection layer refines the encoded features as shown in Equation (5).

(5) $$F_{P}=w_{P}{FR}_{P}\left(F_{L}\right)$$

where ${FR}_{P}\left(F_{L}\right)$ is the final feature representation from Performer using $F_{L}$, $w_{P}$ is a projection weight matrix, and $F_{P}$ is the extracted features using the LeViT-Performer model.

### 2.4. Tensor Fusion-Based Feature Fusion

**Figure 5:**
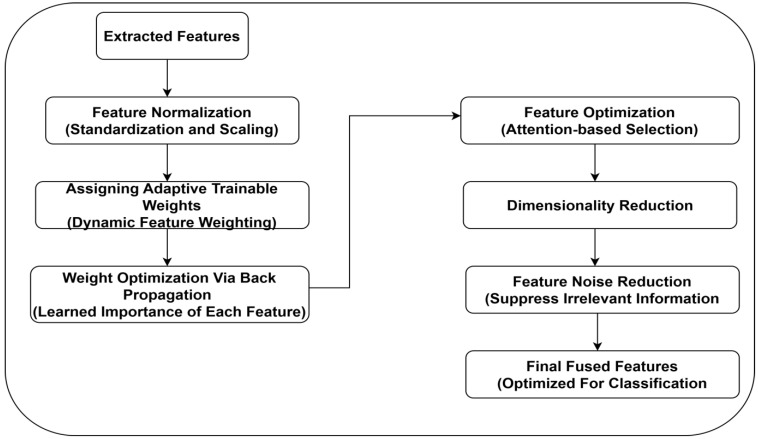
Feature fusion approach.

Effective integration of multi-source feature representation is essential for achieving high classification accuracy. In order to retain local and hierarchical feature representation, the authors employed a tensor fusion approach [27,28]. Unlike conventional feature fusion approaches, tensor fusion uses adaptive weights. It allows the model to prioritize relevant features of ischemia and infection classes. Dimensionality reduction is used to remove redundant features, reducing computational overhead. By leveraging this approach, memory footprint can be reduced, enabling real-time implementation with limited resources. Figure 5 presents the suggested feature fusion approach.

The authors applied feature normalization to prevent feature dominance. The feature maps were extracted from different architectures with distinct dimensions. The authors applied a learnable projection transformation to ensure compatibility. Equation (6) presents the normalization of feature maps.

(6) $$\left\{\begin{array}{c} F_{M}=\mathrm{Normalization}\left(W_{M}F_{M}\right)\\ F_{S}=\mathrm{Normalization}\left(W_{S}F_{S}\right)\\ F_{P}=\mathrm{Normalization}\left(W_{P}F_{P}\right) \end{array}\right.$$

where $W_{M}$, $W_{S}$, and $W_{P}$ are learnable weight matrices, projecting feature maps into a common latent space to guarantee dimensional compatibility before fusion.

The trainable fusion weights (α, β, and γ) are used to dynamically adjust the contributions of each feature. Equation (7) shows the feature fusion process.

(7) $$F_{fused}=\alpha \;F_{M}+\beta F_{S}+\gamma F_{P}$$

where $F_{fused}$ is the fused feature, $F_{M}$ is the MobileNet V3 features, $F_{S}$ is the SWIN features, $F_{P}$ is the LeViT-Performer features, and α, β, and γ are adaptive fusion weights optimized through gradient descent. These weights are normalized to ensure stability, as shown in Equation (8).

(8) α+β+γ=1, α, β, γ ≥0.

Equation (9) highlights the loss function, optimizing fusion weights for accurate DFUs classification.

(9) $$L_{fused}=\sum_{i=1}^{N}{\left\|y_{i}-f\left(F_{fused}\right)\right\|}^{2}$$

where $y_{i}$ is the ground truth label, $f\left(F_{fused}\right)$ is the outcome after feature fusion, and *N* represents the number of samples.

Furthermore, the authors applied substantial dimensionality reduction using principal component analysis (PCA) to minimize the computational overheads.

### 2.5. Ensembled Splines Driven KANs-Based Classification

The KANs [29] classifier utilizes three splines, B-splines, natural splines, and polynomial splines, to classify ischemia and infection classes. Each spline provides a different level of smoothness and flexibility in decision boundaries. An ensemble voting mechanism is applied to combine the outcomes of three splines, achieving a stable classification. B-splines provide smooth and stable approximations. It can handle subtle variations in ulcer shape and color. Natural splines identify gradual variations in ulcer texture better than B-splines. Polynomial splines fit high-order polynomials to data, capturing ulcer region complexity. A voting-based ensemble mechanism is used to improve prediction accuracy and generalization. The authors employed a soft voting (weighted voting) approach to reduce the impact of single misclassification. Equation (10) presents the voting approach for classifying the images.

(10) $$P_{F}\left(y=\left(C_{j}|F_{fused}\right)\right)=\sum_{i=1}^{N}w_{i}P_{i}\left(y=\left(C_{j}|F_{fused}\right)\right)\;$$

where $P_{F}$ is the final prediction, y is the class label, $C_{j}$ is the class with probability ($P_{i}$), and $w_{i}$ is the weight assigned to classifier *i* based on BOHB optimization.

To interpret the feature contributions, the SHAP [30] value is used. It analyzes the importance of each fused feature. Equation (11) outlines the computation of the SHAP values.

(11) $$S_{i}=Z\left[KANs\left(F_{fused}\right)\right]-Z\left[KANs\left(F_{fused}\setminus i\right)\right]$$

where $S_{i}$ is the SHAP values of different splines, $Z\left[KANs\left(F_{fused}\right)\right]$ is the prediction with all features, and $Z\left[KANs\left(F_{fused}\setminus i\right)\right]$ is the prediction when feature i is removed.

## 3. Results

The proposed model was developed and evaluated in a high-performance computing environment to ensure efficient training and inference. The experiment was performed on Windows 11 Pro (64-bit), powered by an Intel i7+ processor with NVIDI GeForce RTX 3080 Ti (12 GB GDDR 6X VRAM). The tensor cores of the RTX 3080 Ti were used to optimize matrix operations, batch normalizations, and self-attention mechanisms. The authors implemented the model using Python 3.9., providing a stable ecosystem for feature extraction and classification. The TIMM (Version 0.6.12.) library was employed for MobileNet V3-SWIN and LeViT-Performer-based feature extractions. In addition, different libraries, including Torchvision (version 0.12.0), NumPy (version 1.21.25.), Albumentations (version 1.1.0.), MatPlotlib (version 3.5.1.), and Seaborn (version 0.11.2.), were used for the model development. Table 2 highlights the configuration details for the model implementation.

The authors evaluated the model’s performance using well-structured dataset partitioning and comprehensive evaluation metrics, ensuring robustness and statistical reliability. The DFUC 2021 dataset was divided into three subsets: train set (70%), validation set (15%), and test set (15%). To assess the generalization capability across different datasets, the DFUC 2020 dataset was utilized, using 20% of its images. This strategy was used to guarantee that the model was not overfitted to a single dataset. The performance metrics, including accuracy (Acc), precision (Prec), Recall (Rec), F1-score (F1), and specificity (Spec), were used to determine the model’s classification ability. The area under the receiver operating characteristic curve (AUROC) is used to ensure clinical reliability and real-world applicability. In addition, standard deviation (SD) and confidence interval (CI) are analyzed to quantify model consistency.

**Figure 6:**
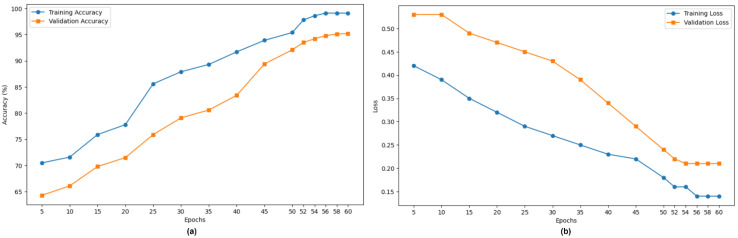
(**a**) Training and (**b**) validation analysis.

Figure 6a,b highlight the accuracy and loss progression over 60 epochs, reflecting the model’s convergence behavior. Figure 6a indicates a consistent convergence rate without overfitting. The convergence behavior reflects the well-optimized training process. The absence of a widening gap between the training and validation losses ensures that the model learns patterns without overfitting or underfitting. Feature normalization, dropout, or weight regularization support the model in reducing overfitting and minimizing the training and validation losses.

Table 3 underscores the model’s state-of-the-art performance in classifying images across datasets. The findings of the DFUC 2020 dataset reinforce the model’s generalization capabilities, maintaining a high accuracy of 97.2% for ischemia and 96.7% for infection. The consistent performance demonstrates the model’s generalization, reliability, and clinical applicability in a real-world environment. The multi-scale representation enhances the potential of the proposed model in determining ischemia and infection features.

**Figure 7:**
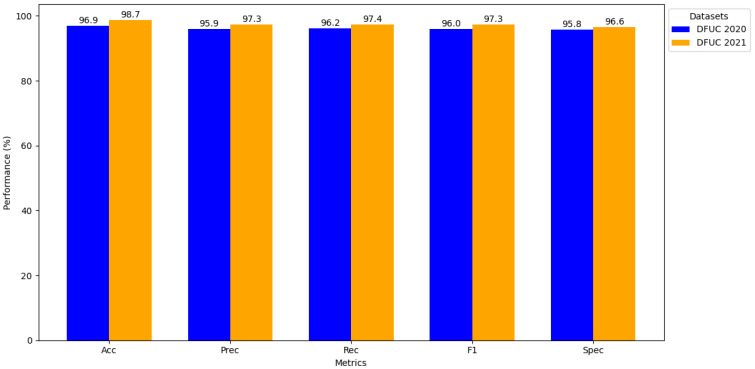
Findings of the performance evaluation.

Figure 7 displays the comparative results of the proposed model across DFUC 2020 and DFUC 2021 datasets. It highlights the robustness and effectiveness of the proposed model in DFUs classification. The model achieves an average accuracy of 98.7% on DFUC 2021, indicating consistent and improved classification performance. F1-Score of 97.3% (DFUC 2021) and 96.0% (DFUC 2020) reflects balanced precision and recall scores. The optimal feature representation and robust classification model are the factors for the successful DFUs classification.

The authors systematically evaluated the impact of each component on classification performance to validate the effectiveness of the fusion process. Table 4 reveals the findings of the ablation study. The removal of feature extractors leads to a significant drop in accuracy, showing the contribution of local, hierarchical, and hybrid features. The elimination of tensor fusion and usage of direct concatenation reduces accuracy by 3.6%. This indicates that trainable fusion dynamically balances feature contributions effectively. The replacement of KANs with a standard fully concatenated layer results in an accuracy drop of 4.1%, demonstrating the KANs model’s significance.

Table 5 provides a detailed evaluation of the proposed model on the DFUC 2021 dataset. Compared to baseline and individual models, the proposed model achieves the highest accuracy, 98.7%, with a low SD of 0.0004 and a narrow CI of 96.3–97.1. Comparatively, SWIN and Linformer achieve better accuracy. However, the limitations in the feature extraction reduced their overall performance. The pre-trained models were ideal for larger datasets, affecting their recall and specificity on domain-specific datasets. The innovative feature extraction and classification render the proposed model suitable for real-world DFUs classification.

**Figure 8:**
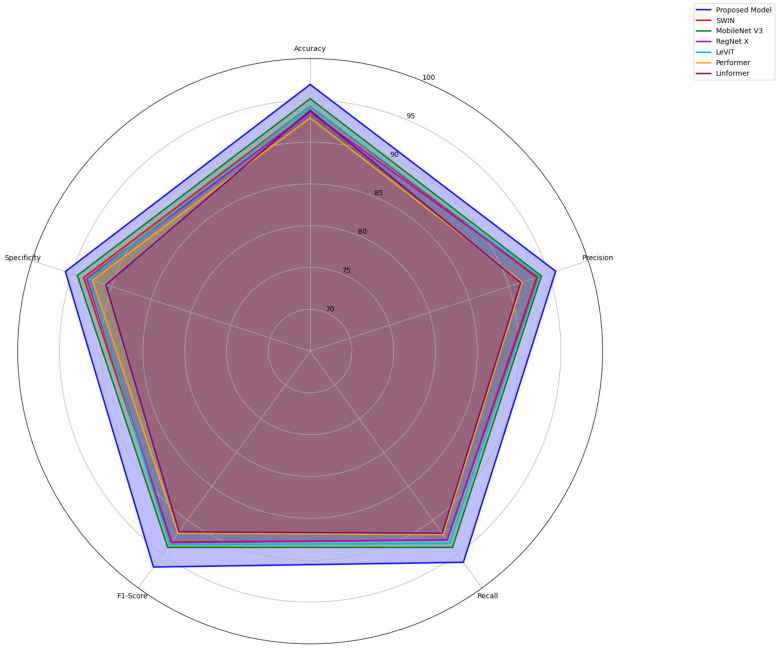
Findings of the comparative analysis—DFUC 2020.

Figure 8 reveals the comparative analysis of the proposed DFUs classification against baseline models. It highlights the superior generalization across standard evaluation metrics. The specificity of over 95% underscores the potential of the proposed model in identifying positive cases. The suggested tensor fusion produced compact and informative features, enhancing generalization to novel data. Regularization strategies prevent model overfitting during training, contributing to successfully classifying ischemia and infection cases. In contrast, the pre-trained models lack advanced attention mechanisms, causing challenges in detecting unique patterns of DFUs.

Table 6 illustrates the findings of the specific classes with respective SHAP values. SHAP 1 offers local spatial details, capturing ulcer textures, color, and edge to the prediction. SHAP 2 presents the hierarchical contextual relationships, including surrounding tissue conditions or ulcer size. SHAP 3 represents the combination of local and global features, exhibiting overlapping characteristics of ischemia and infection. A higher SHAP value for a specific feature region indicates that the feature strongly influences the prediction. In contrast, a negative SHAP value represents that the feature reduces the likelihood of the prediction.

**Figure 9:**
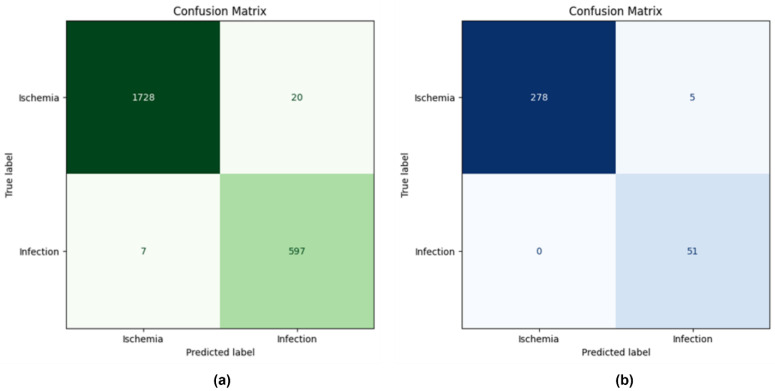
Confusion matrix (**a**) DFUC 2021 (**b**) DFUC 2020.

Figure 9a,b demonstrate the model’s ability to classify ischemia and infection cases accurately. The matrices provide insights into the number of correctly and incorrectly classified samples. In clinical settings, false positives (incorrectly classifying a non-severe ulcer as severe) lead to unnecessary interventions, such as aggressive treatments or hospitalization, increasing healthcare costs and patient distress. Likewise, false negatives (failing to detect a severe ulcer) may result in infection, gangrene, and potential amputation. By being trained and validated on clinically evaluated datasets, the model predictions inherently reflect expert medical assessments, rendering it suitable for real-world deployment. The proposed model integrates adaptive fusion, fine-tuned KANs, and SHAP-based interpretability, reducing false positives and negatives. The absence of false negatives indicates the model’s sensitivity, covering critical DFUs cases. The low number of false positives and negatives across datasets presents the model’s balanced performance. The robustness of the proposed EL approach in handling diverse clinical data yielded minimal misclassification rates.

**Figure 10:**
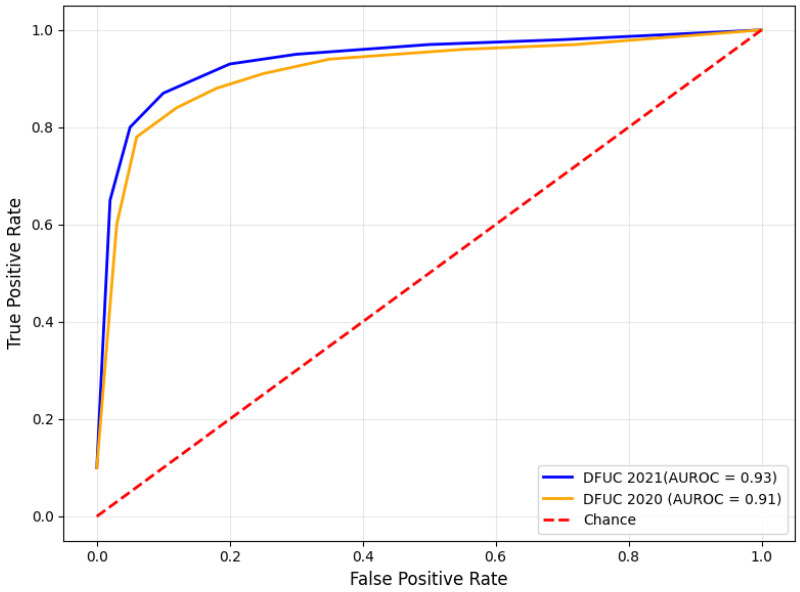
Findings of the AUROC analysis.

Figure 10 presents the AUROC curves for DFUs classification. The results demonstrate the significance of the model in balancing true positive and false positive rates. The near-perfect AUROC values reflect the remarkable classification performance of the proposed model. The findings guarantee the model’s adaptability and generalization capability. The consistent AUROC values across DFUC 2020 and DFUC 2021 datasets highlight the model’s adaptability to variations, such as differences in image resolution, lighting, and ulcer complexities.

Table 7 presents the effectiveness and superiority of the proposed model compared to existing approaches. The built-in interpretability addresses the limitations in existing models, making it a valuable tool for clinical applications. The AUROC values showcase the exceptional discriminative power of the proposed model. The existing studies [31,32,33,34] obtained lower accuracy due to the limited feature extraction capabilities. Toofanee et al. (2022) [35] achieved an accuracy of 95.0% on the DFUC 2021 dataset. In contrast, the proposed model maintains superior performance across datasets, indicating robust generalization. Previous models, including those presented by Qayyam et al. (2022) [36] and Sarmun et al. (2024) [37], obtained lower F1, demonstrating challenges in handling class imbalances. The dynamic integration of multi-source features and effective modelling of non-linear relationships enables the proposed model to deliver exceptional outcomes. The combination of advanced techniques through the innovative approach supports the proposed model in addressing the shortcomings of existing methods, such as lower performance, lack of robustness, and limited interpretability.

## 4. Discussion

This study proposes a unique DFUs classification model, improving feature extraction, fusion, and interpretability. The model delivers state-of-the-art performance through the integration of advanced techniques, including MobileNet V3, SWIN Transformer, LeViT-Performer, tensor fusion, and ensemble splines-based KANs. According to the findings of comparative analysis, the suggested techniques improve the model’s resilience, interpretability, and reliability. One of the novel contributions of this study is the hybrid feature extraction framework. The combination of CNNs and ViTs allows the model to capture global and local features. In order to differentiate ischemia and infection, MobileNet V3-SWIN captures subtle patterns, including ulcer margins, granulation, texture patterns, adjacent tissue conditions, and extensive inflammatory regions. LeViT-Performer offers hybrid feature representations integrating the benefits of CNNs and transformers to uncover spatial and global relationships. This multi-source feature extraction addresses the shortcomings of pre-trained models emphasizing either local or global features.

The comparison analysis demonstrates that the suggested model outperforms current methodologies, attaining accuracies of 98.7% on DFUC 2021 and 96.9% on DFUC 2020. These findings demonstrate its capacity to generalize across datasets, guaranteeing reliability in clinical applications. The combination of hybrid feature extraction, adaptive tensor fusion, interpretable and explainable KANs-based classification, and robust generalization strategies enable the proposed model to achieve state-of-the-art classification accuracy, outperforming existing models in terms of accuracy, interpretability, and real-world applicability. The key factor for the successful classification is tensor fusion. Unlike concatenation and early fusion techniques, tensor fusion uses adaptable and trainable weights to integrate the extracted features dynamically. By using this strategy, the model optimizes the hierarchical and local characteristics of DFUs images. This dynamic and efficient fusion technique supports the model’s exceptional performance, illustrating its efficacy in managing unbalanced classes and varied DFUs representations. The standalone CNNs encounter challenges with global context awareness, leading to misclassification in complex DFU cases. In contrast, the application of hybrid feature extraction and KANs-based DFUs classification supported the proposed model to encode hierarchical relationships and long-range dependencies, enhancing DFUs classification.

The use of ensemble splines-based KANs for classification is an additional distinctive characteristic of this research. KANs classification allows the model to approximate decision boundaries accurately. It guarantees clinical relevance and strong classification performance, in contrast to the existing DFUs classification model. Employing adaptive spline functions in KANs improves model interpretability, revealing the impact of individual characteristics on predictions. The interpretability of DFUs classification is crucial in therapeutic applications. Aligning with clinician demands and promoting trust, the model clearly explains its predictions by including SHAP values in the pipeline. The findings of the comparative analysis reinforce the significance of the proposed model’s capability in classifying DFUs images. The proposed model surpassed the existing approaches by achieving superior performance across different evaluation metrics. It can deliver reliable and adaptable real-time solutions for DFUs classification, supporting clinicians in diagnosing and managing DFUs with confidence.

The inference time of the proposed DFUs classification model depends on different factors, including hardware configurations and computational optimization. On a high-performance GPU, the model’s average inference time per image is ~30–50 ms. The inference time increases to ~220–570 ms on a CPU-only system. These outcomes indicate the capability of the model to deliver real-time predictions when deployed on GPU-enabled environments, making it suitable for real-time diagnostic workflows. The model can be integrated into electronic medical records and telemedicine settings, assisting clinicians in rapid DFUs management. Incorporating it on telemedicine platforms can reduce the need for frequent in-person visits. This strategy can enable seamless communications between patients, general practitioners, and wound care specialists, facilitating comprehensive DFUs management.

The model can be hosted on cloud platforms, supporting healthcare providers to upload DFUs images through a secure online portal. Using this approach, individuals in remote locations can receive expert medical opinions without visiting healthcare centers. The deployment of the model in mobile health applications can allow patients to capture DFUs images using their smartphones, leading to early-stage DFUs assessment. The SHAP value interpretability reveals that the model addresses adversarial attacks. The proposed MobileNet V3-SWIN-LeViT-Performer-based feature extraction framework with tensor fusion incorporates several mechanisms that improve the proposed DFUs classification model’s resilience to adversarial perturbations. The combination of local and global features reduces sensitivity to pixel-level perturbations. Reducing redundancy in feature extraction prevents an adversarial attack from misleading the model. The tensor fusion strategy maintains the model’s resilience through the dynamic modification of feature weights. The trainable fusion weights prioritize reliable features when an adversarial attack or noise affects feature channels.

To mitigate potential biases, several key approaches were incorporated into the proposed model. The model was trained and validated on DFUC 2021 and 2020 datasets. The cross-dataset validation indicates the model’s generalizability across multiple settings. The pre-trained weights of MobileNet V3, SWIN, LeViT, and Performer benefit the model from a broader feature representation before fine-tuning using the DFU-specific features. The SHAP-value-based interpretation allows the model to verify whether ulcer predictions were based on clinically relevant areas rather than dataset-specific biases. A direct comparison between the proposed DFUs classification and clinician-based diagnosis can be analyzed in terms of accuracy, consistency, detection of subtle features, and inference time. Clinicians use visual inspection, patient history, and clinical tests to classify DFUs. Subjective clinical evaluations may overlook subtle patterns of ischemic and infection. In addition, clinicians require substantial time to assess an ulcer. In contrast, the proposed model achieves an accuracy of 98.7% using DFUs images in limited inference time, superior to inter-rater variability among human experts.

The authors encountered several challenges during the model development. Integrating diverse features requires dimensionality reduction and batch-wise processing in order to reduce memory overhead. To maintain a balance between local and global features, careful tuning of fusion weights is required. This approach prevents overemphasis on specific feature types. In addition, advanced regularization techniques and data augmentation strategies are essential to handle class imbalances in the DFUs datasets. Implementing the proposed model in a resource-constrained environment may face challenges due to the complexities of the tensor fusion strategy and ensemble splines-based KANs classification. The use of adaptive splines provides a high-level understanding of the decision-making process. This limitation could hinder the adoption of the model in clinical workflows. The reliance on a single modality may not fully capture the complexities of DFUs diagnosis. The dependency on pre-trained models may limit the adaptability to entirely novel datasets or unseen clinical variations. The suggested modular approach may lead to suboptimal performance on highly diverse datasets. Substantial training is required to improve the overall model’s performance. Despite the high classification accuracy, certain misclassifications may occur due to inherent challenges in DFUs imaging. Ischemic ulcers may have subtle color variations, reducing the model’s ability to differentiate the ulcers from normal skin tones. Lighting inconsistencies may obscure vascular deficiencies. This limitation may affect the model in capturing microvascular patterns associated with ischemia detection. The absence of diverse ethnic and demographic representations may reduce the applicability of the proposed model to diverse patient populations. Salam et al. [39] demonstrated the importance of integrating diverse demographic data, including ethnicity and age, into DL models to enhance their robustness and generalizability. The training data encompassing diverse demographic variables can enhance the model’s effectiveness. Using these data, the proposed model can learn to recognize a wider range of ulcer manifestations. This strategy can improve the model’s clinical applicability.

By quantifying the contribution of each feature, SHAP values provide a deeper analysis of feature importance. In a clinical setting, the value highlights specific ulcers, tissue textures, and vascular structures, influencing the predictions of ischemia or infection. The longitudinal DFUC datasets ensure the stability of the proposed model across diverse datasets, improving real-world adoption. To enhance SHAP’s clinical utility, higher-order feature interactions can be incorporated in order to provide context-aware explanations. Using visual heatmaps, clinicians can understand the model’s decision-making process. This transparency can enable model validation, ensuring its alignment with expert diagnostic patterns. Validating the proposed model across diverse clinical settings can guarantee its real-world applicability. By following the findings of Khan et al. [40], the model’s generalizability can be improved. Multi-center data benchmarking can be performed to maintain the model’s performance across different imaging sources. Novel test sets can be used to ensure better generalization. Data privacy is a significant challenge in medical AI development. Asif et al. [41] discussed the significance of the federated learning framework for secure model generalization. The federated learning framework can facilitate secure model generalization. By adopting federated learning, the proposed model can generalize across diverse clinical settings without compromising data privacy.

The authors utilized TensorRT for NVIDIA GPU in order to deploy the proposed model in resource-constrained environments. The utilization of linearized self-attention reduces computational overhead while maintaining feature extraction efficiency. The integration of MobileNet V3-SWIN and LeViT-Performer may pose challenges for deployment in resource-constrained environments, such as mobile devices and edge computing systems. Future research avenues can extend the applicability and interpretability of the proposed model. Developing lightweight architectures for tensor fusion and KANs can optimize computational efficiency. The integration of pruning strategies with the proposed approach can enable model deployment in resource-constrained clinical settings. By applying model pruning, redundant or less significant weights can be systematically removed without affecting performance. Quantization can be used to convert the model parameters from floating-point to lower-bit representations, reducing memory usage and computational costs. Low-rank decompositions can be employed in ViTs architecture to minimize computational complexity. Enriching the dataset with early-stage ulcers and mixed ischemia and infection samples can improve the model’s adaptability. The inclusion of counterfactual explanations or attention heatmaps can enhance the model’s transparency and acceptance in clinical environments.

## 5. Conclusions

This study introduces a novel and innovative paradigm for treating and managing DFUs. It addresses key challenges in detecting and classifying DFUs severities. The integration of novel feature extraction and classification outperforms existing state-of-the-art approaches. The study findings highlight the strength and adaptability of the proposed model, providing a valuable tool for early and accurate DFUs severity classification. By leveraging SHAP values, the proposed model offers insights into feature importance, allowing clinicians to understand the model’s decision. This transparency strengthens the model and supports healthcare’s demand for explainable healthcare solutions. The generalization performance on the DFUC 2020 dataset demonstrates the potential of the proposed model on unseen data. It underscores the model’s ability to handle the complexities of DFUs classification. However, the model has certain limitations. The dependency on pre-trained models may require additional fine-tuning in real-time implementation. The computational demands of tensor fusion and KANs classification may cause challenges in deploying the proposed model in resource-constrained environments. Future avenues of this research should focus on incorporating multi-modality data to provide a holistic understanding of DFUs severities. Further advancements in explainability can offer deeper insights into the model’s decision-making process, enhancing clinical trust.

## Figures and Tables

**Table 1:** Dataset features supporting model’s interpretability.

Feature Name	Description	Importance For Model’s Interpretability
Ulcer shape	Geometric structure of the ulcer	Distinguishing ischemia from infection
Ulcer texture	Granulation tissue pattern, surface roughness, and irregularities	Differentiating healthy and ulcer patterns
Ulcer color	RGB distribution of ulcer region	Distinguishing necrotic and infected ulcers
Wound exudate	Presence of fluid in the ulcer	Infection indicator
Depth information	Shallow or deep ulcers based on color gradients	Significant for assessing ulcer severities
Lighting condition	Brightness, contrast, and exposure variation	Ensuring model robustness under clinical image variability
Foot region	Forefoot, mid-foot, and heel ulcer region	Significant for making clinical decisions

**Table 2:** Computational strategies.

Model	Parameters	Values
MobileNet V3- SWIN	Backbone	MobileNet V3
	Transformer head	SWIN transformer with windowed self attention
	Batch size	64
	Optimizer	AdamW
	Learning rate	$1\;\times \;10^{-4}$
	Weight delay	$1\;\times \;10^{-2}$
LeViT-Performer	CNN-Transformer fusion	LeViT combines CNN’s spatial efficiency with Performer optimized self-attention
	Self-attention	Linearized attention
	Activation	GELU
	Batch size	32
	Optimizer	SGD
	Learning rate	$5\;\times \;10^{-4}$
	Dropout	0.3
Tensor fusion-based fusion	Feature attention scaling	Weighted sum with trainable co-efficient
	Feature reduction	PCA
KANs-based classification	Hyperparameter tuning	BOHB
	Regularization	0.007
	Number of hidden nodes	64
	Learning rate	$4.8\;\times \;10^{-4}$
	Batch size	32
	Splines functional order	3rd order splines
	Output layer	Sigmoid

**Table 3:** Performance analysis.

	Acc	Prec	Rec	F1	Spec
DFUC 2021					
Ischemia	98.6	97.1	97.3	97.2	96.5
Infection	98.9	97.5	97.6	97.5	96.7
DFUC 2020					
Ischemia	97.2	95.8	96.3	95.9	95.9
Infection	96.7	96.1	96.1	96.0	95.8

**Table 4:** Ablation study (DFUC 2021).

Model Variant	Acc	Prec	Rec	F1	Spec
Proposed Model	98.7	97.3	97.4	97.3	96.6
Without LeviT-Performer and Fusion (MobileNet V3-SWIN + KANs)	96.4	94.5	94.9	94.7	94.1
Without MobileNet V3-SWIN and Fusion (LeViT-Performer + KANs)	95.8	93.9	94.2	94.0	93.7
Without Tensor Fusion (MobileNet V3-SWIN + LeViT-Performer + Concatenation + KANs)	95.1	93.1	92.8	93.0	92.1
Without KANs (MobileNet V3-SWIN + LeViT-Performer + Fusion + Fully Connected Layer)	94.7	92.5	92.3	92.4	91.8

**Table 5:** Comparative analysis—DFUC 2021.

	Acc	Prec	Rec	F1	Spec	SD	CI
Proposed Model	98.7	97.3	97.4	97.3	96.6	0.0004	[96.3–97.1]
SWIN	93.8	93.4	93.7	93.5	93.9	0.0003	[96.1–96.8]
MobileNet V3	94.9	94.1	94.3	94.2	94.1	0.0005	[95.3–95.9]
RegNet X	92.1	91.3	90.8	91.0	90.2	0.0005	[95.8–96.7]
LeViT	93.7	92.4	91.9	92.1	91.4	0.0005	[96.1–97.4]
Performer	93.1	92.9	93.4	93.1	93.1	0.0003	[96.3–96.7]
Linformer	94.5	93.6	94.1	93.8	93.9	0.0003	[95.9–96.4]

**Table 6:** Sample outcomes.

Input Image	Ground Truth	Prediction	SHAP Value	Clinical Relevance
** 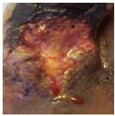 **	Infection	Infection	0.35, 0.45, and 0.20	Highlighted regions indicate redness, swelling, and potential pus, leading to infection-specific patterns.
** 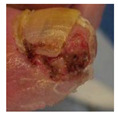 **	Infection	Infection	0.40, 0.50, and 0.10	Yellowish discharge and wound edges are the crucial indicators of infection. In addition, swelling and wound patterns influenced the prediction.
** 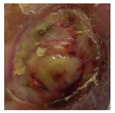 **	Infection	Infection	0.38, 0.47, and 0.15	Central ulcer discharge and edge discoloration indicate a bacterial infection.
** 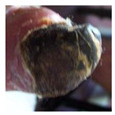 **	Ischemia	Ischemia	0.45, 0.40, 0.18	Dark necrotic tissue and reduced blood supply areas confirm ischemic interpretation.
** 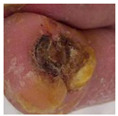 **	Ischemia	Ischemia	0.50, 0.35, and 0.11	Yellowish dry tissue with no surrounding swelling, indicating ischemia diagnosis.
** 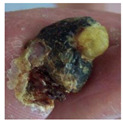 **	Ischemia	Ischemia	0.42, 0.16, and 0.37	Shrunken tissue regions represent the ischemia patterns.

**Table 7:** Findings of the comparative analysis.

**Model**	**Dataset**	**Feature Extraction**	**Classification**	**Interpretability**	**Performance**
Proposed Model	DFUC 2021 [22]	MobileNet V3-SWIN and LeViT-Performer	Ensembled Splines-based KANs	✓	Acc: 98.7% Prec: 97.3% Rec: 97.4% F1: 97.3% Spec: 96.6% AUROC: 0.93
Proposed Model	DFUC 2020 [22]			✓	Acc: 96.9% Prec: 95.9% Rec: 96.2% F1: 96.0% Spec: 95.8% AUROC: 0.91
AlGarawi et al. (2022) [31]	DFUC 2020 [22]	Customized CNNs	Fully connected layer	×	Average Acc: 86.7% Average F1: 86.7% AUC: 0.90
Galdran et al. (2022) [32]	DFUC 2020 [22]	ResNeXt 50—EfficientNet-ViT-based DeiT	Fully connected layer	×	Average Prec: 70.0% Average Rec: 68.5% Average F1: 75.7% AUC: 0.88
Bloch et al. (2022) [33]	DFUC 2021 [22]	EfficientNet B0-B6	Fully connected layer	×	Average F1: 53.5% Average Prec: 68.5% Average Rec: 66.5% AUC: 0.86
Ahmed and Naveed (2022) [34]	DFUC 2021 [22]	EfficientNet B0-B6—ResNet 50	Fully connected layer	×	Average F1: 53.9% Average Prec: 67.3% Average Rec: 67.1% AUC: 0.59
Toofanee et al. (2023) [35]	DFUC 2021 [22]	EfficientNet-BeiT	KNN-based classification	×	Acc: 95.0% AUC: 0.8298 Prec: 93.9% Rec: 93.9% F1: 93.7%
Qayyam et al. (2022) [36]	DFUC 2021 [22]	Customized ViTs	Fully connected layer	×	Average F1: 43.47% Average Prec: 68.0% Average Rec: 66.3% AUC: 0.84
Sarmun et al. (2024) [37]	DFUC 2021 [22]	YOLO V8X	Fully connected layer	×	Prec: 89.7% Rec: 74.0% F1: 81.1%
		YOLO V8X + ResNet	EL approach	×	Prec: 83.5% Rec: 75.2% F1: 79.1%
Ahsan et al. (2023) [38]	DFUC 2020 [22]	ResNet 50	Fully connected layer	×	Average Acc: 92.12% Average F1: 92.24% AUC: 0.87

Note: ✓ (Presence of Model’s interpretability) and × (Absence of Model’s interpretability).

## Data Availability

The Diabetic Foot Ulcers Dataset is publicly available https://dfu-challenge.github.io/dfuc2021.html, accessed on 22 October 2024.
